# Translational neuroscience measures of fear conditioning across development: applications to high-risk children and adolescents

**DOI:** 10.1186/2045-5380-3-17

**Published:** 2013-09-01

**Authors:** Tanja Jovanovic, Karin Maria Nylocks, Kaitlyn L Gamwell

**Affiliations:** 1 Department of Psychiatry and Behavioral Sciences, Emory University School of Medicine, 49 Jesse Hill Jr Dr, Suite 331, Atlanta, GA 30303, USA

**Keywords:** Anxiety disorders, Development, Childhood, Adolescence, Fear conditioning, Extinction

## Abstract

Several mental illnesses, including anxiety, can manifest during development, with onsets in late childhood. Understanding the neurobiological underpinnings of risk for anxiety is of crucial importance for early prevention and intervention approaches. Translational neuroscience offers tools to investigate such mechanisms in human and animal models. The current review describes paradigms derived from neuroscience, such as fear conditioning and extinction and overviews studies that have used these paradigms in animals and humans across development. The review also briefly discusses developmental trajectories of the relevant neural circuits and the emergence of clinical anxiety. Future studies should focus on developmental changes in these paradigms, paying close attention to neurobiological and hormonal changes associated with childhood and adolescence.

## Review

### The importance of translational approaches for anxiety disorders

Anxiety disorders, such as specific phobias and social anxiety, are highly prevalent, and can develop early in life and be severely disabling [1]. Although fears in childhood are common and normative, they may become pathological if they interfere with function or extend later than the normal developmental pattern. In a replication of the National Comorbidity Survey anxiety diagnoses were found to be highly prevalent at 28.8% and the earliest disorder to emerge with a median age of 11 years [2]. From the developmental perspective, anxiety disorders that emerge in adolescence may be impacted by hormonal changes associated with puberty [3], or with neuroanatomical changes during brain development [4]. Several longitudinal studies of children and adolescents found no sex differences in childhood, but a highly significant increase in anxiety disorders in girls relative to boys in adolescence [5].

Given this complexity, progress in the field can be greatly enhanced by using models of anxiety based on neuroscience. Translational approaches use laboratory paradigms developed in animal models to investigate neurobiological phenotypes of clinical phenomena [6]. For instance, fear conditioning offers a unique framework for translational studies, given that responses to danger are highly conserved across species and can therefore be modeled in animal experiments. Conceptualizing anxiety disorders within this framework affords the use of laboratory paradigms, such as fear conditioning and fear extinction, to better understand altered fear processing and to develop better treatment and prevention programs for anxiety disorders [6]. Given that the groundwork in fear neurocircuitry has been greatly developed in animal models, human clinical research can capitalize on these findings [7]. This review will describe and summarize findings from animal and human neuroscience across different developmental stages and discuss their relevance for the ontogeny of anxiety disorders.

### Paradigms for the study of fear and anxiety from basic science

#### Fear conditioning

Fear conditioning is based on a simple Pavlovian conditioning model in which a neutral conditioned stimulus (CS), for example, a light, is paired with an aversive unconditioned stimulus (US), for example, electric shock. After a number of pairings, the association is formed so that the CS alone elicits the conditioned response (CR), for example, freezing in rodents or fear-potentiated startle in humans [8]. This basic model is used in animal as well as human research to investigate mechanisms of fear expression [9-14]. Conditioning can be accomplished using several stimulus modalities as the CS: in animal research the primary cues have been auditory [10] or olfactory [15,16], whereas human studies have typically used visual stimuli [14]. A recent human study using auditory cues found that this modality also lends itself well to fear conditioning in people [17]. There have also been applications of different types of aversive US. Animal studies have almost exclusively used electric shock; however, human studies have included more diverse stimuli, such as air blast to the larynx [11,17,18], audio files of a woman screaming [19], loud noises [20], and aversive muscle contractions [21]. The alternatives to electric shock have also produced robust fear conditioning, without the increased anticipatory anxiety of shock delivery [19]. These less aversive types of US have been especially useful with more sensitive participants, such as those in clinical or pediatric research.

**Figure 1:**
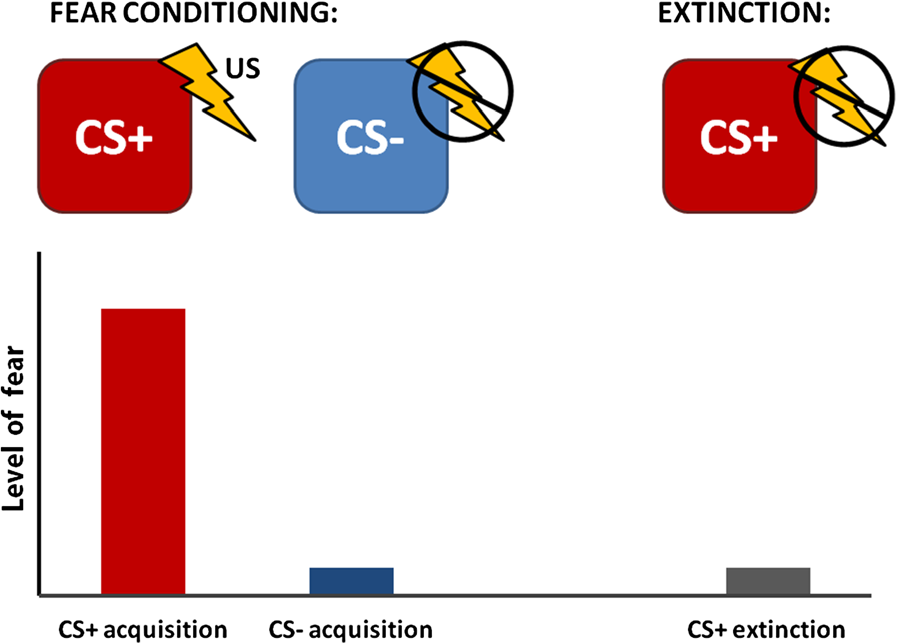
**Schematic representation of fear conditioning and extinction showing experimental design (top panel) and expected outcomes with regard to level of fear (bottom panel).** CS, conditioned stimulus; US, unconditioned stimulus.

There are two basic fear conditioning paradigms: a single cue paradigm in which the CS is reinforced by the US (the CS+, sometimes referred to as a ‘danger signal’), or a differential conditioning paradigm in which one CS is reinforced, while a different CS is never paired with the US (the CS-, sometimes referred to as a ‘safety signal’). Figure 1 shows a schematic of fear conditioning and the expected outcomes. The reinforcement schedule of the CS+, that is, the percentage of CS trials that are paired with the US, can vary from 100% to as little as 30%. Although successful fear conditioning can be accomplished with such small percentages, they usually take more trials. Human research in fear conditioning often uses a CS+ and a CS-, with the difference between the two frequently used as the index of the conditioned response [22,23].

The advantage of using these paradigms is that they can be measured with peripheral outcomes such as the skin conductance or startle responses, which are noninvasive but offer physiological measures of fear conditioning. Fear conditioning is also highly translational, in that very similar tests can be used across different animal species, including rodents [10,24], non-human primates [25,26], and humans [11,22,27]. In fact, fear-potentiated startle, which can be measured with the acoustic startle reflex in the presence of a fear-conditioned CS, can be assessed in all mammalian species [28]. Some fear responses that have been used in fear conditioning paradigms, such as skin conductance response (SCR), are only measured in humans, whereas others, such as freezing behavior, are primarily measured in animals. Regardless of the specific responses measured, the benefit of this paradigm is that several decades of animal research have clearly established the neural bases of fear conditioning and the circuitry and molecular mechanisms are very well understood. This research has indicated that the amygdala, located in the brain limbic circuit in the temporal lobe, is necessary for fear-conditioned responses [10,24,29].

#### Extinction

Extinction is another commonly used paradigm based on Pavlovian conditioning. It follows a fear-conditioning (or fear-acquisition) experiment in which a CS is reinforced with an aversive US. In fear extinction paradigms, the stimulus that was previously paired with the US (that is, the CS+) is then repeatedly presented without the US, so that it no longer elicits a fear response [30-32] (see Figure 1). Whereas fear acquisition refers to learning that something is dangerous, extinction is a mechanism by which an individual learns that something that was previously dangerous has become safe. Most research has supported the theory that extinction involves new learning processes [33] rather than erasure of the fear memory. However, recent data suggest that in some cases, erasure may also be occurring [34,35]. The reinforcement schedule during fear acquisition can affect the rate of extinction, in that 100% reinforcement results in faster extinction, whereas lower percentage reinforcement can prolong extinction [32]. In some cases where facilitation of normal rates of extinction is being tested either pharmacologically or behaviorally, a lower reinforcement schedule allows room for improvement during extinction. Another method for achieving suboptimal extinction is by providing fewer extinction trials; this approach also has the advantage of requiring less time during both acquisition and extinction, which can be a significant factor in the design of human research. The same types of CS described above for fear conditioning have been used with extinction. Similarly, the same conditioned responses, that is, fear-potentiated startle, SCR, and freezing, have been used in extinction studies.

An important distinction to make with regard to extinction is differentiating within-session extinction, referring to the decrease in fear responses that occur during a single extinction session from between-session extinction, which refers to the retention of low fear responses on a separate occasion using the same CS [36]. Within-session extinction is also referred to as extinction training, since this is the phase during which new learning about the CS/US contingency occurs. Between-session extinction is also called extinction recall (or extinction test), since it requires activation of the previously learned memory of the CS/US contingency at some time after learning. Extinction tests most frequently occur 24 hours after extinction training and are highly context dependent, inasmuch as differences in experimental context will result in a return of the fear response (termed renewal [37]). An extinguished fear response can also return with the presentation of unpaired US’s (termed reinstatement [31,38]), or simply with the passage of time (termed spontaneous recovery [8]). These phenomena lead to the discovery that the original fear memory is not erased during extinction, but rather replaced with new learning [39]. As is the case with fear conditioning, extinction has been well-studied in animal models and its neurobiological underpinnings include the amygdala, as well as the hippocampus and the prefrontal cortex [32,40].

### Animal fear conditioning studies across development

#### Infant and juvenile period

**Figure 2:**
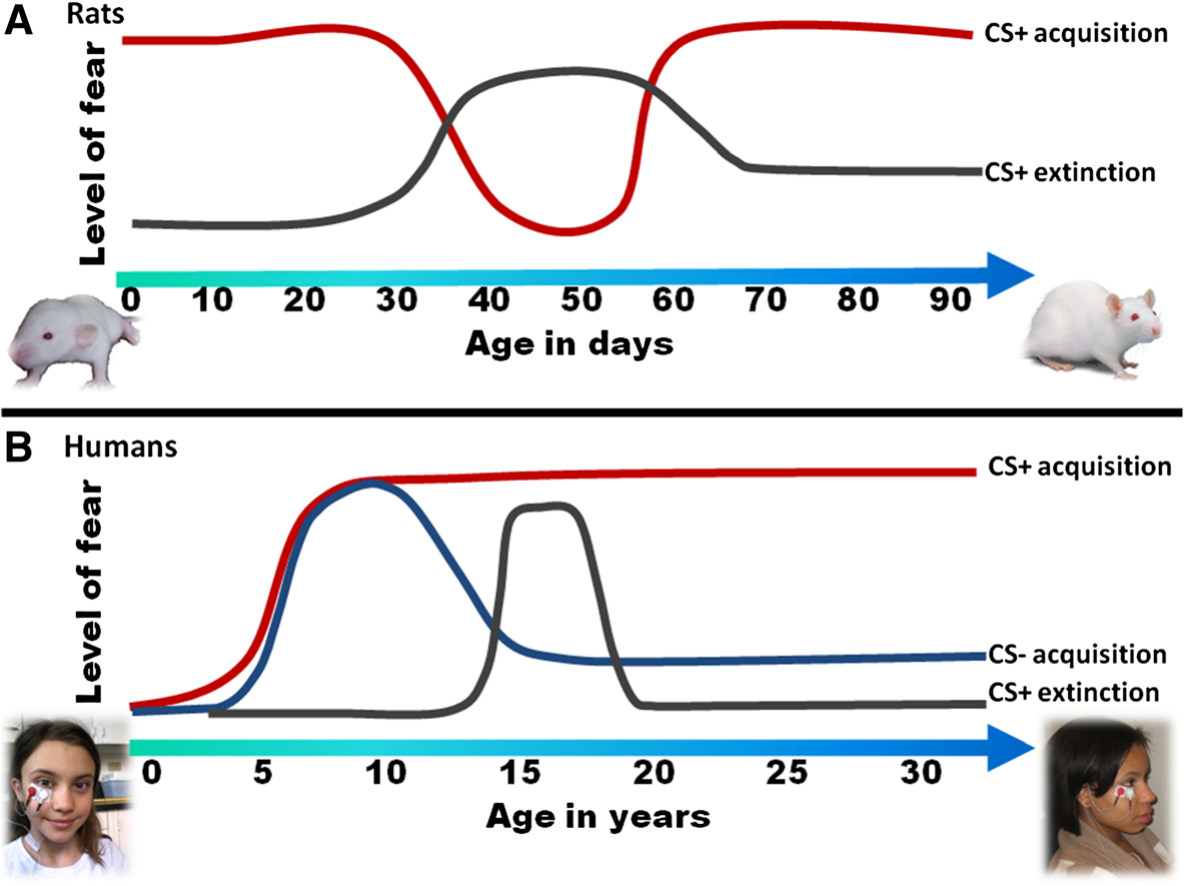
**Diagram of changes in levels of fear during fear conditioning and extinction across development in rats (top panel, A), and humans (bottom panel, B).** CS, conditioned stimulus. Informed consent provided for photographs.

Although comparing developmental changes between species has inherent limitations, some parallels can be drawn between animal and human studies. Figure 2a shows a schematic of fear conditioning and extinction across age in rodents. Most rodent studies include altricial species, such as rats and mice; in these species the neonates are born without fur, unable to move, and their vision develops postnatally. However, olfactory stimuli can be perceived at birth. For this reason, the studies using the youngest subjects have focused on olfactory fear conditioning [16,41]. Using olfactory cues is a highly ecologically valid approach, given that the infants’ survival depends on recognizing maternal odors. Classical conditioning experiments using olfactory cues as the CS and electrical shock as the US in rat pups up to postnatal (PN) day 8 have found that the association that is formed leads to approach rather than fearful behavior towards the conditioned odor [16]. On the other hand, if rats are fear-conditioned after PN day 9, they develop adult-like responses, that is, they avoid the olfactory cue that was paired with the shock [41]. The authors argue that painful stimuli in very young infants may signal maternal behavior, such as stepping on the pups, and thus lead to approach behavior. Developmentally, rat pups begin to walk around 9 days of age and explore outside the nest; at this stage it is of crucial importance that the infants learn to discriminate between dangerous and safe conditions [16].

In a series of elegant experiments, Sullivan and colleagues described the biological mechanisms for the switch from approach to avoidance learning. In young neonates, the pairing of the odor with shock activates the olfactory bulb, but not the amygdala [16]. The amygdala is activated only after PN day 9 by the input of corticosterone, the stress hormone, which is released in response to the shock at this stage of development. Amygdala activation during odor-shock pairing results in the adult-like fear-conditioned response. Interestingly, the fear-conditioned response can be elicited in pups at PN day 8 and younger if corticosterone is administered; conversely, it can be delayed after PN day 9 if the mother is present during conditioning, since her presence suppresses the release of endogenous corticosterone [41]. After weaning age (PN days 21 to 23), maternal presence no longer suppresses adult-like fear-conditioned responses [16]. This may also be the age at which the hippocampus begins to store fear-conditioned information. Early studies of context versus cue conditioning found that rats at PN day 18 show CS-specific fear conditioning, but not context conditioning [42]. On the other hand, rats at PN day 24 show both forms of conditioning. Since context conditioning is hippocampally mediated [42], these data suggest that the amygdala develops earlier in the juvenile period than the hippocampus.

Another group of researchers has focused on fear extinction in infant and juvenile rat pups [43], using both auditory and olfactory cues during fear conditioning. In a series of experiments, Richardson and colleagues demonstrated that the neural mechanisms underlying extinction in infant rats (PN day 16) is fundamentally different from those in juvenile rats (PN day 24). At both ages, the amygdala is involved in fear conditioning and fear expression, as assessed by freezing behavior in the presence of the CS+; however, the prefrontal cortex is involved in fear extinction only in the older age group [44]. As described above, fear extinction in adults involves new learning, which inhibits the fear response via the medial prefrontal cortex (mPFC) and the hippocampus [39]. However, given that the mPFC and hippocampus are late-maturing structures [45], in infant rats extinction is solely amygdala-dependent [34] and seems to result in erasure of the original memory in the amygdala [43]. Support for this hypothesis is demonstrated by the lack of context conditioning [42] and context-dependent fear renewal [46] in rats under age PN day 18, which was observed in rats PN day 23 and older, and the absence of fear reinstatement upon presentation for an unpaired US in the infant group [47].

#### Adolescent period

As mentioned above, rat pups are weaned at 3 weeks of age and transition to adolescence at PN day 35. Mice show similar, but slightly more rapid development, transitioning to adolescence at PN day 29 [48]. In an extensive study of the effects of age on fear conditioning, Pattwell and colleagues conditioned and tested mice in separate age groups 2 days apart, starting with age PN day 23 through PN day 39. They found that juveniles at ages PN days 23, 25, and 27 showed adult-like levels of fear conditioning, as expected. However, adolescent mice at PN days 29, 31, and 33 showed significantly reduced levels of fear (see Figure 2a). This difference appeared to be limited to the expression on fear, rather than fear learning; mice that were conditioned at PN day 29 demonstrated normal fear responses when tested two weeks later [48], suggesting that the fear conditioning occurred but was suppressed during adolescence.

With regard to fear extinction, similar findings have been shown in mice and rats indicating a lack of fear extinction during adolescence. Mice at PN day 29 demonstrated decreased extinction training (that is, within-session extinction), but also less extinction retention between sessions [49]. Another study of adolescent rats at PN day 35 found the same effect, that is, reduced extinction in PN day 35, while younger rats at PN day 24 showed adult-like levels of extinction [50] (Figure 2a). Interestingly, neurons in the inhibitory region of the mPFC (infralimbic cortex) show activity after extinction in juveniles and adults, but are inactive in adolescents [50]. This suggests that the decrease in extinction was not due to neural development, but that inhibitory circuits may be quiescent during this period. It is possible the amygdala is undergoing changes in synaptic inputs from the thalamus during this period [51], which could explain both the reduced expression of conditioned fear and the lack of extinction.

Aside from rodent studies, there are very few studies in other non-human species investigating fear conditioning across development. Most studies using non-human primates have used observational learning paradigms, such as watching a monkey express fear of snakes [52], or fear-potentiated startle [26] in adult monkeys. Although paradigms assessing anxious behavior, such as the human intruder paradigm have been used in younger animals [53], only a few studies have examined fear conditioning in juvenile monkeys. One such study found significant increases in startle response to the CS+ in 2-year-old rhesus macaques, with those who were separated from their mothers immediately after birth showing greater fear-potentiated startle compared to mother-reared animals [54]. The study concluded that early stress was associated with increased fear responses. Since early-life stress is a known risk factor for adult psychopathology, including anxiety disorders and depression [55], this study is a good example of how controlling early environment in animal research can contribute to the understanding of causal factors for human anxiety.

### Implications for human clinical research

The above studies provide several useful avenues for translational research. First, they point to sensitive periods in neuronal development that affect fear conditioning. Even with the caveat of species differences in rates of maturation, onset of reproductive function provides a reference point to compare across these different mammalian orders. The developmental trajectories indicate that amygdala nuclei are the earliest to develop, followed by the hippocampus and then the mPFC. In younger animals, fear memories are more labile as they appear to rely entirely on the amygdala for expression, whereas, adolescence is marked by changes in fear expression and deficits in extinction.

Second, animal models can provide a basis for more detailed analysis of underlying mechanisms. For example, a genetically modified mouse for the brain-derived neurotrophic factor (*BDNF*) gene shows abnormal secretion of BDNF from neurons and anxiety-like behavior [56]. Importantly, these anxious phenotypes observed in this BDNF mouse model begin to develop during pre-puberty, and are associated with the estrous cycle [57]. Carriers of the risk allele for BDNF, the Met form of the Val66Met polymorphism, show alterations in fear conditioning and extinction deficits both in rodents and humans [58]. Furthermore, this same BDNF polymorphism is associated with increased amygdala activation in adolescent humans with anxiety disorders when viewing fearful stimuli. Although a review of the genetic and molecular mechanisms of fear conditioning is outside the scope of this paper (we refer the reader to Mahan and Ressler, 2012 for a recent review [59]), this example illustrates the power of translational neuroscience approaches to clinical research. In the following sections, we will review the human developmental literature and the association between anxiety and fear conditioning during the childhood and adolescent periods.

### Human fear conditioning studies across development

#### Childhood period

Figure 2b shows a schematic of fear conditioning to danger signals, fear inhibition to safety signals, and fear extinction across age in humans. Fear conditioning in children has a long history, starting with Watson’s famous experiment, in which baby Albert was conditioned at nine months of age to be afraid of a white rat by pairing its presentation with a fear-eliciting loud noise [60]. However, there is a small body of data investigating psychophysiological measures of fear conditioning across development. Due to the translational focus of this review, we will discuss only those studies that included psychophysiological data such as startle and skin conductance. The startle reflex can be measured in very young children; one study examined startle in infants from 2 to 6 months of age, and found a gradual increase in startle magnitude over that age range [61]; another study found that 5-month-old infants showed modulation of startle with emotion [62]. An affective modulation of the startle study with children from 3 to 9 years old and adults found an increase in baseline startle with age, but equivalent levels of affective modulation across all groups [63]. An early classical conditioning study in children from 2 to 11 years of age suggested that healthy children did not show discrimination between CS+ and CS- stimuli prior to 6 years of age [64]. This study was partially replicated in a recent study of skin conductance responses during fear conditioning with children at ages 3 through 8 years [65]. The study found that the fear conditioned responses increased with age, with a large increase between 5 and 6 years, which then reached a plateau. Development of fear-potentiated startle was investigated in older children, between 8 to 13 years, using faces as the CS and a scream as the US [66]. This study found that fear-potentiated startle to the CS+ was greater in the 10- to 13-year-old group compared to the 8- to 9-year-old group. Furthermore, the study suggested that age 10 years may be critical in inhibiting fear responses to safety cues, that is, children in the 8 to 9 years age group showed higher responses to the CS- and poor generalization between the CS+ face and a generalization stimulus face that was a 50/50 morph between the CS+ and CS-. We have recently found the same age effect on discrimination between danger and safety signals [67], with fear reduction to the CS- emerging at age 10 years (see Figure 2b). There are two studies of extinction in the literature, which examined skin conductance responses in healthy children: the first study included children between 8 and 12 years old [20], and the second tested extinction in 5- to 11-year-old children [49]; both studies found normal (adult-like) levels of extinction to the CS+. Although no studies have specifically examined fear extinction across normal development using startle, one study indicates that healthy children (ages 7 to 13 years) show a reduction in fear-potentiated startle to the CS+ during extinction, which is paralleled with SCR and fear ratings [68].

#### Adolescent period

As mentioned above, few studies have focused on the developmental effects of puberty on fear conditioning, so most have grouped children with adolescents. However, some specifically focused only on adolescents [69-71], or separately analyzed data from children (ages 5 to 11 years) and adolescents (ages 12 to 17 years) [49]. Unlike the rodent studies described above, the human data suggest that adolescents show normal levels of fear conditioning and adult-like fear responses post acquisition (Figure 2b). In all studies in which a danger cue (CS+) was compared to a safety cue (CS-), adolescents showed increased fear-potentiated startle [69,70] and skin conductance responses [49,71] to the danger cue. However, there may be similarities between rodents and humans in fear extinction during adolescence. The only study to directly compare extinction across species during childhood and adolescence found reduced extinction in adolescents compared to both children and adults [49], see Figure 2b. The results of this study indicated that there may be a reduction in extinction during this developmental stage due to a lack of synaptic plasticity in the PFC. It is also possible that hormonal changes during puberty impact extinction, as data from animal and human studies in adults suggest that estrogen levels play a role in extinction via modulation of the mPFC [72,73].

### Associations between clinical anxiety and fear conditioning in children and adolescents

A very small number of studies have examined the effect of anxiety and depression on fear-conditioned responses in children. Waters and colleagues included anxious and non-anxious children between 8 and 12 years of age in their study of fear conditioning, using a loud tone as the US [20]. The results indicated that anxious children showed greater fear responses to all types of CS during conditioning and extinction compared to controls, and did not discriminate between danger (CS+) and safety (CS-) signals on SCR. Another study using a similar loud noise US paradigm with startle and SCR found that anxious children showed deficits in extinction to the CS+, that is, fear-potentiated startle was higher in anxious compared to non-anxious children [68]. We have also found that anxiety was associated with decreased inhibition of fear-potentiated startle to safety signals in children [67]. Similar findings were shown in a fear-potentiated startle paradigm that used an airblast as the US in 8- to 12-year old children with high and low levels of depressive symptoms. In this study depression was positively correlated with startle to the danger cue, but not the safety cue [74].

Fear conditioning has also been shown to be associated with anxiety in adolescents. Adolescents at high risk for anxiety due to having a parent with anxiety show elevated startle responses during fear conditioning [69]; similarly, adolescents who were rated behaviorally inhibited as children and have current anxiety, show higher fear-potentiated startle to the danger cue [70]. The effect of anxiety was even more pronounced to the safety cue, that is, inhibition of fear to the safety cue was significantly impaired in behaviorally inhibited adolescents with high anxiety. A recent longitudinal study examined startle during danger and safety cues in high school students and found that startle responses to the safety cue during the baseline assessment in adolescence predicted onset of anxiety disorders during the next 4 years [21]. This association was specific to anxiety disorders, in that unipolar depression was not predicted by startle to the safety cue.

Given that observed sex differences in fear conditioning [75] may emerge in puberty due to activational effects of gonadal hormones, anxiety may affect male and female adolescents differently. A study of high-risk adolescents that examined fear-potentiated startle separately for males and females found that female offspring of adults with anxiety disorders had heightened startle responses to all trial types (that is, baseline, safety and danger cues), whereas high-risk males only showed increased startle to the danger cue compared to low-risk males [69]. A possible interpretation of these data is that the female adolescents showed context conditioning, that is, being tested in the context in which fear conditioning occurred increased startle to all trial types. This type of response is a marker of more non-specific anxiety, and may be dependent on the bed nucleus of the stria terminalis (BNST) rather than the amygdala [76]. The BNST is sexually dimorphic [77] and may be the basis for post-pubertal differences in startle responses [78]. In support of this argument, a recent study using a startle paradigm designed to compare responses to predictable and unpredictable aversive events found that adolescent girls had higher potentiation of the startle response in the unpredictable condition compared to boys [79]. There were no sex differences in the predictable condition, which elicited fear-potentiated startle in all participants. Given that unpredictable aversive events (that is, random delivery of air blasts) would generate non-specific anxiety, this response may also be mediated by the BNST. However, more research is needed with a focus on pubertal effects to delineate development of sex differences.

### Neural bases of fear conditioning in humans: developmental trajectories

In accordance with animal research, brain imaging studies with humans have found that the amygdala modulates the fear response: presentation of conditioned fear cues results in amygdala activation in several studies using positron emission tomography (PET) and functional magnetic resonance imaging (fMRI) [29,80,81]. Neuroimaging studies demonstrate that fear acquisition and extinction of fear also activate the prefrontal cortex, specifically the ventromedial PFC (vmPFC) [32]. Recent developments in the spatial resolution of neuroimaging techniques have resulted in more fine-tuned examinations of this area of the brain. For example, the rostral or subgenual regions of the anterior cingulate cortex (ACC) are activated during the regulation of emotional stimuli [82] including fear stimuli [32,83]. There are several lines of evidence that this region of the vmPFC is associated with inhibition of fear: fMRI data indicate increased activation during extinction recall after extinction learning [32,84]. Activation of this area during a fMRI response-inhibition task is also correlated with inhibition of fear-potentiated startle to safety signals [85].

In order to better understand the neurobiology of developmental changes in fear conditioning responses, we will briefly review human developmental milestones, with a special emphasis on the amygdala, hippocampus and the mPFC, given the importance of these structures for fear conditioning. Early studies using structural MRI [86] showed that amygdala volume increased in male individuals from ages 4 to 18 years, whereas hippocampal volume increased in female individuals in the same age range. More recent studies have found significant effects of age and sex on these subcortical structures, but not an interaction effect [87]. On the other hand, cerebral gray matter develops in a quadratic trajectory (inverted U-shaped curve), showing early increases in volume and thickness, followed by decreased volume and density after adolescence [4,87,88]. More specifically, grey matter volume and thickness in the prefrontal cortex decreases from adolescence to adulthood [89]. Total cerebral volumes peak in late childhood to early adolescence with female individuals reaching this peak about 4 years earlier than male individuals before starting to decline, so that male individuals on average have 9 to 12% larger volume compared to female individuals [90]. White matter continues to increase after adolescence [91], with the mPFC showing the longest developmental trajectories [92]. Emerging data suggest that the development of the white matter tract is associated with puberty [93]. These sex-specific changes are likely due to differences in receptors for gonadal steroids [86].

A very small number of studies have investigated developmental trends in activity in the above neural structures. One study examined fMRI during fear conditioning using the screaming lady US paired with faces as the CS in adolescents and adults, and found that compared to adults, the CS+ evoked greater responses in the amygdala and hippocampus relative to the CS- in adolescents [71]. Although no other studies specifically examined fear conditioning using fMRI in children and adolescents, several studies have used fear-relevant cues, such as fearful faces, to activate these structures. In one such study, Moore and colleagues [94] performed longitudinal fMRI scans and behavioral measures on children at ages 10 and 13 years. The study examined brain activation to faces displaying different emotions across the two time points, specifically focusing on the association with pubertal development. The results indicated that pubertal development was associated with greater activity in the amygdala and the PFC to affective stimuli. Furthermore, this fMRI study showed a stronger relationship between emotional stimuli and amygdala activity in participants who had reached adolescence, as compared to pre-adolescent participants. A similar finding was observed using fMRI of socially relevant stimuli with children and adolescents [95]. In this study the amygdala showed higher activation to African American faces relative to European American faces in adolescents but not in younger children. Finally, a recent study found a developmental shift in functional connectivity between the amygdala and the mPFC during the viewing of fearful faces. The cross-sectional study included children from 4 years of age to adults and found that these areas were positively connected prior to age 10 years, and negatively connected after age 10 years [96]. It is interesting that this shift maps onto the age of improved fear inhibition and discrimination between danger and safety signals in the fear-conditioning studies described above [66,67]. The observed negative functional connectivity continued to increase from adolescence to adulthood. Earlier studies using similar methods found that adolescents showed greater amygdala reactivity to fearful faces than adults [97]. Together, these structural and functional data point to developmental decreases in activation in limbic subcortical structures in response to fear-related cues from childhood to adulthood. In healthy children and adolescents, this decrease is paired with increases in inhibition of these structures by prefrontal cortical areas involved in regulation of fear responses during safe conditions. Below we review studies that have examined these structures in anxious children and adolescents.

### Effects of anxiety on neural structures involved in fear processing

Anxiety disorders are associated with larger amygdala volume in children and adolescents [98], which is not observed in other brain structures. Larger amygdala volumes are also found in children with prolonged maternal deprivation early in life [99]. In an MRI study of orphaned children, those who were adopted prior to 15 months of age had the same amygdala volumes as controls, whereas children adopted after 15 months of age showed increased amygdala volumes later in childhood (tested around 10 years of age). Although this early trauma may increase risk for anxiety disorders in children, the MRI results in the study were not directly related to anxiety, since the relationship remained significant even after exclusion of children with anxiety. In addition to increased amygdala volume, the functional connectivity between the PFC and the amygdala is altered in anxiety. A study of adolescents who had early life stress found that female, but not male individuals, had decreased functional connectivity between these circuits, and that this was correlated with higher anxiety symptoms [100]. A recent study used resting-state fMRI to examine functional connectivity of amygdala subregions (centromedial, basolateral and surface amygdala) in adolescents with generalized anxiety disorder [101]. Although this is a task-free paradigm, meaning that it does not measure connectivity in response to presentation of fear-related cues, it has significant implications for connections between the neural circuits involved in processing these emotions. The study found that anxiety decreased connectivity between the central amygdala and the subgenual ACC, as well as the connectivity between the superficial amygdala and brainstem nuclei. These data suggest that anxiety may disrupt normal developmental trajectories in neural circuits related to fear conditioning [67].

## Conclusions

**Figure 3:**
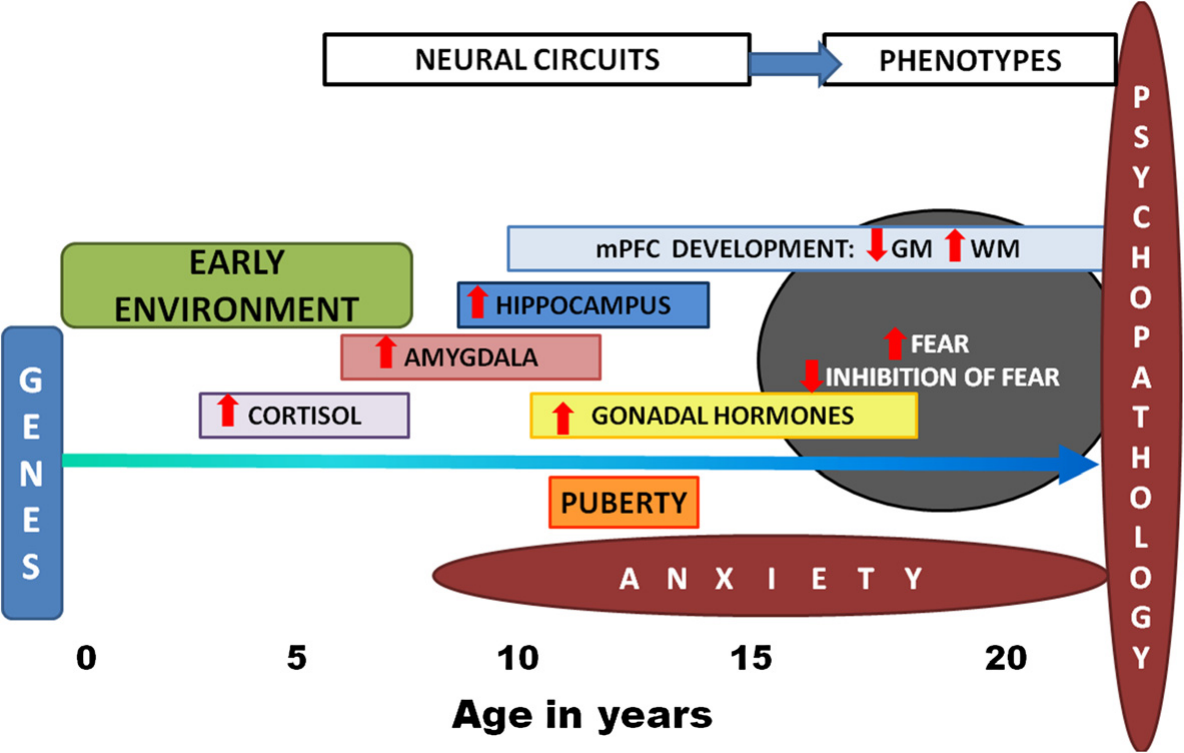
Theoretical model of the effects of genes, environment, and hormonal influences on developmental changes in neural circuits and phenotypes associated with adult psychopathology.

To summarize, the neuroimaging and psychophysiological evidence points to dysregulations in the development of the amygdala and PFC, as well as their connections, as the neural bases for heightened fear responses during fear conditioning and impaired fear inhibition during extinction in children and adolescents at high risk for anxiety disorders. These effects may also differ between male and female individuals; however, these differences may emerge only after puberty. Translational neuroscience models offer a unique opportunity to better understand the neurobiological underpinnings of anxiety disorders through development and puberty. The fear conditioning paradigms described in this review can be used across species and at different stages of development, and provide valuable observable phenotypes. Because they measure outputs of brain circuits associated with fear and anxiety, they are sensitive to the psychopathology of anxiety disorders. Figure 3 shows a theoretical model of the interactions of genetic, environmental, and neuroendocrine factors on neural development and risk phenotypes. Disentangling effects of age from puberty will be important in future approaches aimed at delineating developmental trajectories in healthy and at-risk children and adolescents. In addition to offering insight into abnormalities in these circuits, these paradigms can also point to novel therapeutic targets. The plasticity of fear conditioning and extinction provides a mechanism for early prevention and intervention strategies. Future studies should focus on developmental changes in these paradigms, paying close attention to neurobiological and hormonal changes associated with childhood and adolescence.

## Abbreviations

ACC: Anterior cingulate cortex; BDNF: Brain-derived neurotrophic factor; BNST: Bed nucleus of the stria terminalis; CS: Conditioned stimulus; fMRI: Functional magnetic resonance imaging; PN: Postnatal; mPFC: Medial prefrontal cortex; MRI: Magnetic resonance imaging; PET: Positron emission tomography; PTSD: Post-traumatic stress disorder; SCR: Skin conductance response; US: Unconditioned stimulus; vmPFC: Ventromedial prefrontal cortex.

## Competing interests

The authors declare that they have no competing interests.

## Authors’ contributions

TJ reviewed the literature on psychophysiological paradigms and wrote the first draft of the manuscript; KMN reviewed the literature on brain development and wrote the first draft of the relevant section of the manuscript. KLG reviewed the literature on the development of anxiety disorders and wrote the first draft of that section. All authors revised and approved the final version of the paper.
